# Cisplatin resistant lung cancer cells promoted M2 polarization of tumor-associated macrophages via the Src/CD155/MIF functional pathway

**DOI:** 10.1186/s13046-019-1166-3

**Published:** 2019-04-29

**Authors:** Wen-Chien Huang, Kuang-Tai Kuo, Chun-Hua Wang, Chi-Tai Yeh, Yongjie Wang

**Affiliations:** 1 MacKay Medical College, Taipei, Taiwan; 2 Division of Thoracic Surgery, Department of Surgery, MacKay Memorial Hospital, Taipei, Taiwan; 3 Division of Thoracic Surgery, Department of Surgery, Shuang Ho Hospital, Taipei Medical University, New Taipei City, Taiwan; 4 Division of Thoracic Surgery, Department of Surgery, School of Medicine, College of Medicine, Taipei Medical University, Taipei, Taiwan; 5 School of Medicine, Buddhist Tzu Chi University, Hualien, Taiwan; 6 Department of Dermatology, Taipei Tzu Chi Hospital, Buddhist Tzu Chi Medical Foundation, New Taipei City, Taiwan; 7 Department of Medical Research and Education, Taipei Medical University-Shuang Ho Hospital, Taipei, Taiwan; 8 Department of Thoracic Surgery, The Affiliation Hospital of Qingdao University, Qingdao, China

**Keywords:** Cisplatin resistance, Lung cancer, Cancer stemness, M2 tumor-associated macrophages (M2-TAMs), Src signaling, Multiple kinase inhibitor

## Abstract

**Background:**

Lung cancer often ranks one of the most prevalent malignancies in the world. One of the most challenging aspects of treating late-stage lung cancer patients is the development of drug resistance, from both conventional chemo- and targeted therapeutic agents. Tumor-associated microphages (TAMs) have been shown to promote the survival and distant metastasis of lung cancer cells.

**Methods:**

This study investigated the TAMs - modulating potential of cisplatin-resistant non-small cell lung cancer (NSCLC) cell lines, A549R and H460R by using bioinformatics approach, immunoblotting, immunofluorescence staining, migration, invasion, colony, lung sphere formation and xenograft tumorigenecity assays.

**Results:**

In this study, we first demonstrated the elevated expression of oncogenic and stemenss markers such as Src, Notch1, macrophage inhibitory factor (MIF) and CD155 in trained cisplatin (CDDP)-resistant A549 and H460 cells (A549R and H460R cells). When co-cultured with TAMs, A549R and H460R cells promoted the M2-polarization in TAMs. In addition, A549R and H460R cells showed an increased self-renewal ability as they formed tumor spheres at higher frequency comparing to their parental counterparts. The increased MIF secretion by the A549R and H460R cells could be suppressed by a multiple kinase inhibitor, dasatinib, which resulted in the decreased of oncogenic network of Src, CD155 and MIF expression. Similarly, dasatinib treatment reduced the M2 polarization in TAMs and suppressed self-renewal ability of the A549R and H460R cells.

**Conclusion:**

In summary, cisplatin resistant lung cancer cells not only showed an increased self-renewal ability but also promoted M2 polarization of TAMs via the secretion of MIF. These findings were linked to the increased Src-associated signaling as dasatinib treatment significantly reversed these phenomena. Thus, kinase inhibitors such as dasatinib may be of potential for treating cisplatin-resistant lung cancer by targeting both tumor and the tumor microenvironment.

**Graphical abstract:**

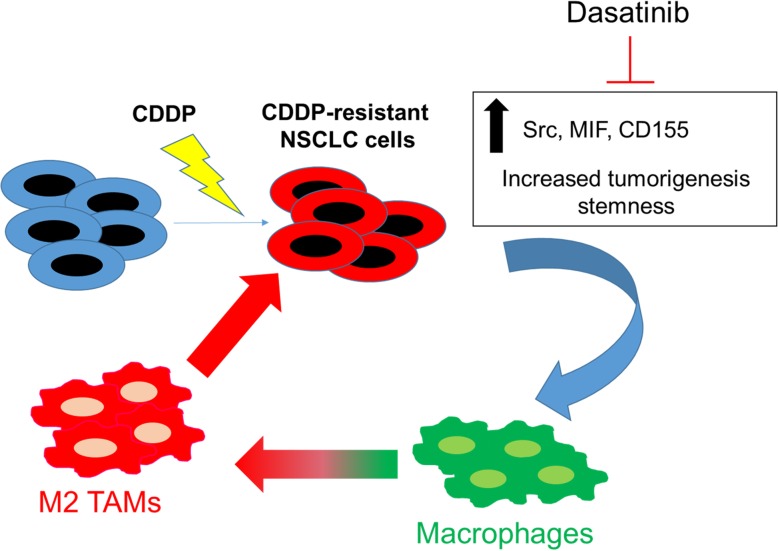

**Electronic supplementary material:**

The online version of this article (10.1186/s13046-019-1166-3) contains supplementary material, which is available to authorized users.

## Background

Despite the advancement in the development of targeted therapeutic agents, lung cancer patients often develop resistance against treatment and disease progression [1]. In the search for more effective interventions, the concept and importance of targeting the tumor microenvironment (TME) has emerged and received much attention. In fact, tumor promoting inflammation (within the TME) has been included in as one of the hallmarks of cancer [2]. As one of the major components of TME, tumor-infiltrating or associated macrophages (TAMs) are responsible for promoting epithelial-to-mesenchymal transition (EMT) and distant metastasis [3]. The polarization of TAMs represents the key of TAMs’ roles in either promoting or inhibitory functions in tumorigenesis. The so-termed M2 TAMs (M2 polarization) are considered to be tumor promoting, characterized by the cytokines produced. It has been shown that there is an intricate cellular communication network established between the cancer cells and M2 TAMs over the course of tumorigenesis. The interruption of the pro-tumor signaling within this tumor-TAM communication network thus represent a potential area for therapeutic development. Thus, finding a common target, which is essential on the survival of tumor cells and sustainability of the tumor microenvironment, is a rational approach. Emerging supports indicate that CD155 could be such target. An elevated serum level of CD155 has been identified in patients of different cancer types [4]. In addition, an elevated level of CD155 was associated to increased metastatic potential [5]. Since M2 TAMs are involved in promoting distant metastasis, we intend to examine the potential link between CD155 expression and M2 TAMs in lung cancer progression.

Cisplatin is perhaps the most prescribed chemotherapeutic agents. Despite its potent cancer-killing efficacy, the development of resistance is almost inevitable. Accumulating evidence suggest different mechanisms responsible for the development of resistance against cisplatin. The generation and maintenance of cancer stem cells (CSCs) have been examined extensively [6, 7]. CSCs have many properties that make them the target for drug development. These properties include self-renewal ability (re-populating tumor mass post treatment), enhanced propensity towards EMT and drug resistance by the increased expression of multiple drug resistance genes. More importantly, TME plays a central role in harboring CSCs. Therefore, in order for an intervention to be effective, both tumor cells and TME must be taken into account.

In this study, we first established cisplatin-resistant non-small cell lung cancer (NSCLC) cell lines, A549R and H460R and demonstrated their increased oncogenic ability and stemness. More importantly, A549R and H460R when co-cultured with TAMs promoted the M2 polarization while M2 TAMs re-enforced the expression of oncogenes and stemness such as Src, Notch1, β-catenin, microphage inhibitory factor (MIF) and CD155. This tumor-M2 TAM communication could be interrupted by the inhibition of Src either via small-molecule agent, dasatinib or gene silencing technique. We showed Src inhibition resulted in the decreased CSCs properties and ability to generate M2 TAMS. Based on our observations, we believe there is a potential for pursuing the use of Src inhibitor in combination with the current chemotherapeutic agents when treating lung cancer patients exhibiting recurrent disease and/or with developed treatment resistance.

## Materials and methods

### Ethics approval and consent to participate

Clinical samples were collected from MacKay Memorial Hospital (Taipei City, Taiwan). All enrolled patients gave written informed consent for their tissues to be used for scientific research. The study was approved by the Institutional Review Board (IRB) of the MacKay Memorial Hospital, consistent with the recommendations of the declaration of Helsinki for biomedical research (Mackay Memorial Hospital approval number: 20160600011 16MMHIS073e) and followed standard institutional protocol for human research. Please refer to Additional file 1: Table S1 and Additional file 2: Table S2 for CDDP-sensitivity determination and clinical characterizations of patients, respectively.

### Cell culture and reagents

Human non-small cell lung cancer (NSCLC) cell lines, A549 and H460 and monocytic cell line, THP-1 were purchased from the American Tissue Culture Collection (ATCC). Cells are maintained under the conditions recommended by the vendor. Cisplatin-resistant A549 and H460 cells were generated according to an established protocol [8] where a continued exposure to escalating concentration of cisplatin (CDDP) over the period of 6 months. Cisplatin [cis-diammineplatinum (II) dichloride] was purchased from Sigma-Aldrich and made into 0.20 M stock solution in NaCl and aliquoted. All cells were acquired or purchased with confirmations for the genotypic authentications from suppliers, and regularly tested for mycoplasma contamination.

### Co-culture and macrophage differentiation

THP-1 cells were treated with 100 nM phorbol-12-myristate-13-acetate (PMA) for 1 day, and the adhered cells were then differentiated into M1 macrophages using 100 ng/ml lipopolysaccharide and 20 ng/ml IFN-γ for 72 h or alternatively differentiated into M2 macrophages using 20 ng/ml IL-4 and 20 ng/ml IL-13 for 72 h, followed by 24 h incubation in RPMI medium in Transwell inserts (pore size, 0.4 μm, Corning, USA). NSCLC cells were then seeded in the lower chamber and co-cultured with M2 macrophages (M0) for 48 h. For dasatinib experiments, both H460R and A549R cells were treated with dasatinib (1 μM, 24 h) prior to co-culturing with M2 macrophages (M0).

### Quantitative PCR reactions

Total RNA of each sample was extracted using a RNeasy Mini kit (QIAGEN, USA). The cDNA templates were generated using an Omniscript RT kit (QIAEN, USA) recommended by the vendor. Reactions were performed using a DNA Engine Opticon (Bio-Rad), and amplifications were performed with the following conditions: total reaction volume: 25 μl, containing 1.5 μl of cDNA, 5 μl of PCR mix, forward and reverse primers at a 0.3 μM final concentration, and SYBR Green (Molecular Probes). Primers used in this study are listed in Table 1. The qPCR results were normalized to those of the housekeeping gene GAPDH.

**Table 1:** Primer sequences for qPCR reactions

Gene	Forward	Reverse
MIF	AGAACCGCTCCTACAGCAAG	TAGGCGAAGGTGGAGTTGTT
CD133	TCCACAGAAATTTACCTACATTGG	CAGCAGAGAGCAGATGACCA
Notch1	CCTGAGGGCTTCAAAGTGTC	CGGAACTTCTTGGTCTCCAG
ABCG2	TCATCAGCCTCGATATTCCATCT	GGCCCGTGGAACATAAGTCTT
ABCB1	AAATTGGCTTGACAAGTTGTATATGG	CACCAGCATCATGAGAGGAAGTC
GAPDH	TGAAGGTCGGAGTCAACGGATT	CCTGGAAGATGGTGATGGGATT
TNF-α	CCTCTCTCTAATCAGCCCTCTG	GAGGACCTGGGAGTAGATGAG
IL-1β	ATGATGGCTTATTACAGTGGCAA	GTCGGAGATTCGTAGCTGGA
CCL22	ATTACGTCCGTTACCGTCTG	TAGGCTCTTCATTGGCTCAG
IL-10	TACGGCGCTGTCATCGATTT	TAGAGTCGCCACCCTGATGT
iNOS	GTTCTCAAGGCACAGGTCTC	GCAGGTCACTTATGTCACTTATC
CCL22	GAGCATGGATCGCCTACAG	CAGACGGTAACGGACGTAATC
Src	GAGGCCCAGGTCATGAAGAA	CCCTTGAGAAAGTCCAGCAAA

### Gene silencing experiments

Src silencing experiments were performed using GIPZ Lentiviral shRNA Transduction Starter Kit and DharmaFECT kb transfection reagent used may require optimization. Three clones were provided: V3LHS_356568; V3LHS_356569; V3LHS_639929. The silencing protocol was carried out according to vendor’s instructions. VC, vector control served as a negative control for the silencing experiments. Src was silenced in both A549R and H460R cells and was validated by Western blots. Anti-Src monoclonal antibody (cat#184Q20, Invitrogen) was used for western blot analysis.

### SDS-PAGE and Western blotting

Cellular protein lysates were isolated using Protein Extraction Kit (QIAGEN, USA), and quantified by Bradford Protein Assay Kit. Equal amount of (20 μg of total protein lysate) each sample was loaded per lane and subjected to SDS-PAGE. Separated proteins were transferred onto a polyvinylidene fluoride (PVDF) membrane and following by a blocking process using a Tris-buffered saline (TBS) plus skim milk. PVDF membranes were then incubated with primary antibodies. All antibodies were purchased from Invitrogen unless otherwise specified. The incubation with primary antibodies was carried out at 4 °C overnight followed by the secondary antibody incubation (room temperature, 1 h). The immunological reaction was detected using chemiluminescent reagent (thermos fisher scientific, USA). All antibodies used for western blotting analysis were purchased from Cell Signaling Technology (CST, USA) unless otherwise specified. Notch 1(#3608, 1:2000); Src (#2109, 1:1000); CD155(#81254, 1:500); β-catenin (#8480, 1:600); MIF (#88186, 1:400).

### Cytokine ELISA assays

Cytokines secreted by macrophages were measured by ELISA assays (LifeSpan BioSciences, USA). The culture media from co-culture experiments with different conditions and treatments (dasatinib treatment or Src-silencing) were collected for analyses. The experiments were carried out according to vendor’s instructions.

### Colony formation assay

To determine the colony forming ability, a soft agar assay was conducted. We used 6-well plates consisting of a base layer of 0.5% agarose gel and an upper layer of 0.35% agarose gel with DMEM/F-12 medium, N2 supplement, 20 ng/mL of EGF, and bFGF. The colony formation efficiency was calculated as the ratio of colony number to the plated cell number.

### Xenograft mouse experiment

The animal study protocol was approved by the Animal Care and User Committee at MacKay Memorial Hospital (Affidavit of Approval of Animal Use Protocol # MMH-014-024). Immune compromised NDO-SCID mice (8 weeks of age, male, 20–25 g of bodyweight) were purchased from the BioLASCO (Taipei, Taiwan). A549R cells (3.0 × 10^6^ cells per injection) were inoculated subcutaneously on the right flank of the mice. Tumor growth was monitored on a weekly basis using a standard hand-held caliper. The tumor volume was calculated by the formula where V (volume) = 0.5 × (a × b^2^); a and b represent the long and short diameters of the tumor, respectively. Mice were humanely sacrificed post experiments and the tumor samples were harvested for further analyses.

### Immunohistochemistry

A standard immunohistochemistry (IHC) protocol was used to analyze tumor samples harvested from the A549R xenograft experiment. Briefly, the sections (5 μm-thick) were first dewaxed and re-hydrated, and the endogenous peroxidase activity was blocked using 3% hydrogen peroxide. Antigen retrieval process was carried out in a pressure cooker where the slides were immersed in 10 mmol/L EDTA (pH 8.0) for 3 min, followed by blocking with 10% normal serum. The sections were the incubated with anti-Src (1:300), anti-MIF (1:200) and anti-CD155 (1:300) antibodies, followed by secondary antibody. The immunostaining was detected by an HRP Polymer Kit (Lab Vision, Fremont, CA, USA). DAB was used as the chromogenic substrate, and sections were counterstained with Gill’s hematoxylin (Fisher Scientific, NJ, USA). The study was approved by the MacKay Memorial Hospital (Approval number: 20160600011 16MMHIS073e) and followed standard institutional protocol for human research. The identity of patients was completely de-identified.

### Online database analyses

Kaplan-Meier survival curves of clinical lung cancer patients from different public databases were generated using online software tool, Kaplan-Meier plotter (http://kmplot.com/analysis/index.php?p=service). This software allows the users to analyze public databases including Cancer Biomedical Informatics Grid (caBIG, http://cabig.cancer.gov/, microarray samples are published in the caArray project), the Gene Expression Omnibus (GEO, http://www.ncbi.nlm.nih.gov/geo/) and The Cancer Genome Atlas (TCGA, http://cancergenome.nih.gov) to identify biomarkers in lung cancer datasets [9].

### Statistical analysis

All experiments were carried out at least three times. Two-tailed t tests were used to analyze the in vitro and in vivo data. The statistical analysis was performed using GraphPad Prism soft-ware where a *p* value < 0.05 was considered as statistically significant and is indicated with an asterisk.

## Results

### Establishment of cisplatin-resistant lung cancer cell lines and the increased stemness

**Fig. 1:**
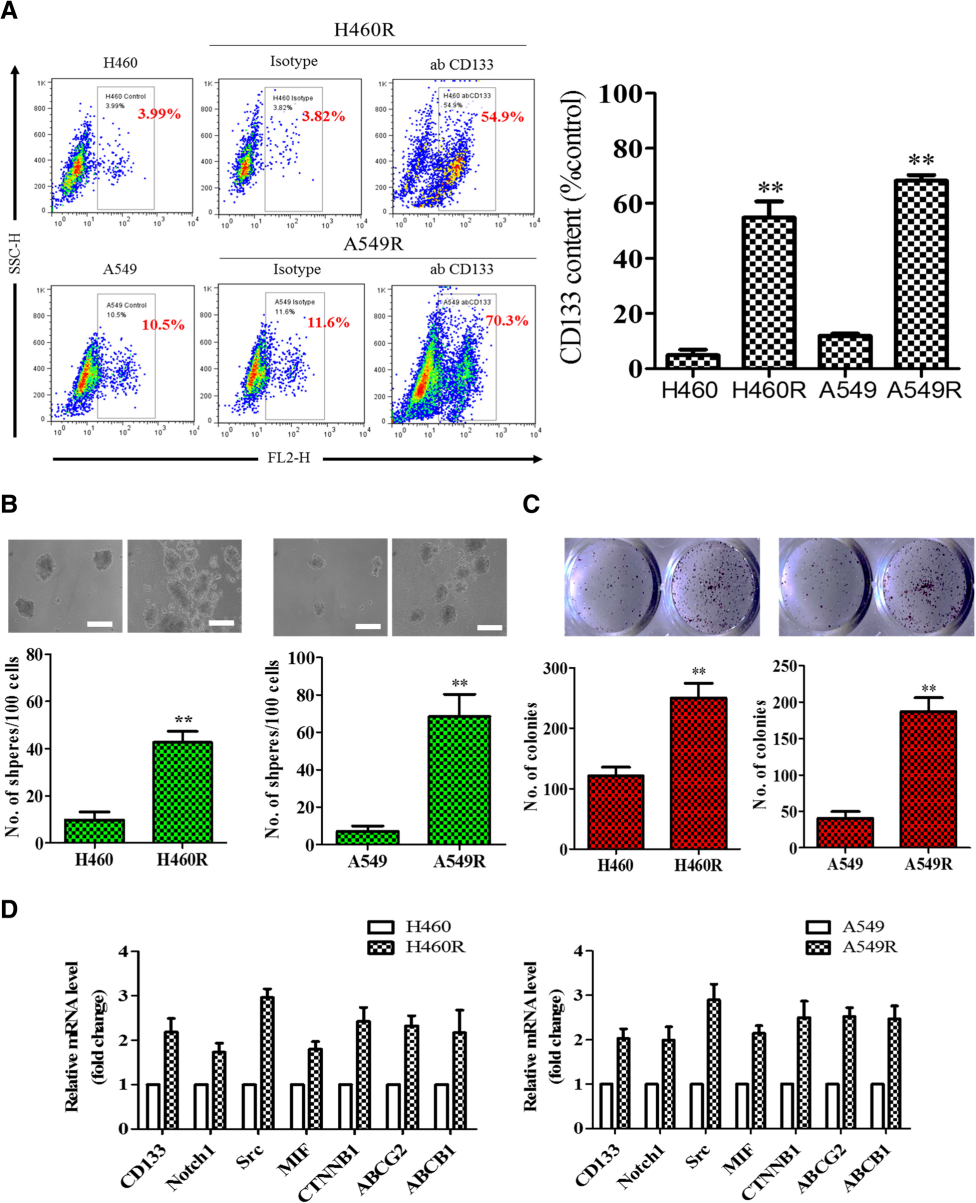
Prolonged cisplatin treatment enriched CDDP-resistant NSCLC cells with increased properties of tumorigenesis and cancer stemness. **a** Flow cytometry analysis showed that a marked increased CD133+ cell population in H460R and A549R cells as compared to their parental counterparts. The bar graph (right) is the quantitative representation of the flow cytometric experiments and showed the comparative level of CD133+ cell population. **b**, **c** Increased tumor sphere and colony forming abilities were also observed in H460R and A549R cells. **d** Real-time PCR profiling of H460R and A549R cells revealed that a significantly increased in oncogenic markers, Src, MIF, stemness markers, CD133, Notch1, β-catenin (CTNNB1) and multiple drug resistant pumps, ABCG2 and ABCB1. *p < 0.05; **p < 0.01; ***p < 0.001

We first tested the notion that cisplatin treatment could lead to the enrichment of drug-resistant NSCLC cells. Human NSCLC cell lines, H460 and A549 cells, were treated with cisplatin for a period of 6 months and the surviving cells were tested for their cisplatin sensitivity. The resistant cells were designated as H460R and A549R cells with a substantially higher IC50 values with respect to their parental counterparts for example, the IC50 value of H460R was found to be greater than 120 μM cisplatin as compared to approximately 37 μM in its parental counterpart (Additional file 3: Figure S1). In addition, the stemness of both H460R and A549R cells were significantly increased as reflected by the increased in the CD133+ cell population (Fig. 1a). CDDP-resistant H460R and H549R cell lines showed approximately 50.9 and 58.7% increase in CD133+ cell population respectively (right bar graph, Fig. 1a). Next, these cells were subject to serum-free culture conditions containing 50 μM CDDP, and we found both H460R and A549R exhibited a significantly higher ability to generate tumor spheres (approximately 4-fold increase in H460R versus H460 cells) in as compared to their parental counterparts, even under high concentration of CDDP (Fig. 1b). Similarly, the colony-forming ability in both cell lines were considerably higher when compared with their parental counterparts (Fig. 1c). For example, H460R formed nearly twice as many colonies as compared with their parental counterparts. We surveyed a panel of markers of cancer stemness and drug resistance in the tumor spheres generated from both parental and CDDP-resistant cells. Expectedly, stemness markers including CD133, Notch1 and β-catenin were significantly upregulated along with oncogenic markers, Src, MIF and drug-resistance genes, ABCG2 and ABCB1 (Fig. 1d). These results showed that a prolonged cisplatin (CDDP) treatment led to the enrichment of NSCLC cells with properties of cancer stem-like cells.

### CDDP-resistant NSCLC cells promoted M2 polarization of macrophages

**Fig. 2:**
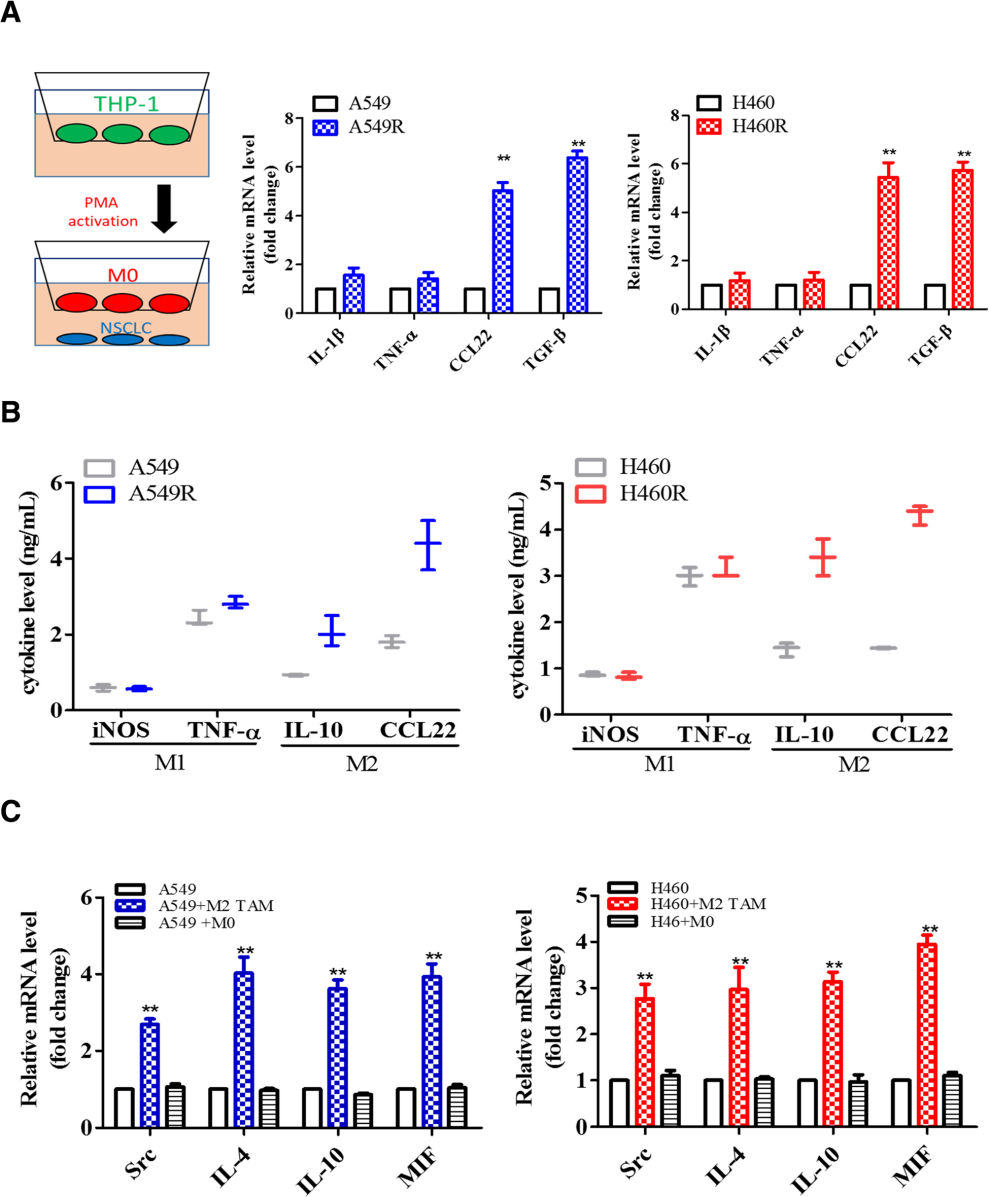
CDDP-resistant NSCLC promoted M2 polarization in TAMs. **a** CDDP-resistant NSCLC cells promoted M2 polarization in THP-1 cells in vitro. The insert depicts the co-culture setup. Real-time PCR analysis demonstrated that macrophages co-cultured with A549R and H460R showed a significantly higher M2 marker (CCL22 and TGF-β) as compared to their parental NSCLC cells. **b** M2 TAMs derived from the co-culture with CDDP-resistant NSCLC cells also showed an increased secretion of M2 cytokines (IL-10 and CCL22) as compared to the TAMs co-cultured with parental NSCLC cells. **c** Real-time PCR analysis demonstrated the increased post TAM co-culture. An increased mRNA level of IL-4, IL-10 and MIF was observed. **p < 0.01; ***p < 0.001

The tumor microenvironment has been recognized to be just as important as tumor cells themselves in driving tumorigenesis [10, 11]. Here, we examined the effects on the polarization of macrophages under the influence of CDDP-resistant NSCLC cells. We observed that both H460R and A549R cells promoted the M2 polarization in THP-1 cells comparatively higher than their parental counterparts (Fig. 2a). M2 macrophage markers including TGF-β and CCL22 were both significantly elevated in THP-1/H460R and THP-1/A549R co-culture as compared to their parental counterparts (Fig. 2a); there was no substantial change in the expression of M1 markers, IL-1β and TNF-α. In support, we detected an increased level of M2 cytokines, IL-10 and CCL22 in the culture medium, while no detectable changes in the level of M1 cytokines, iNOS and TNF-α (Fig. 2b). On the front of cancer cells, both H460R and A549R cells showed a significant increase in the mRNA level of Src, IL-4, IL-10 and MIF as compared to their parental counterparts (Fig. 2c).

### Dasatinib treatment decreased cisplatin-resistance and stemness

**Fig. 3:**
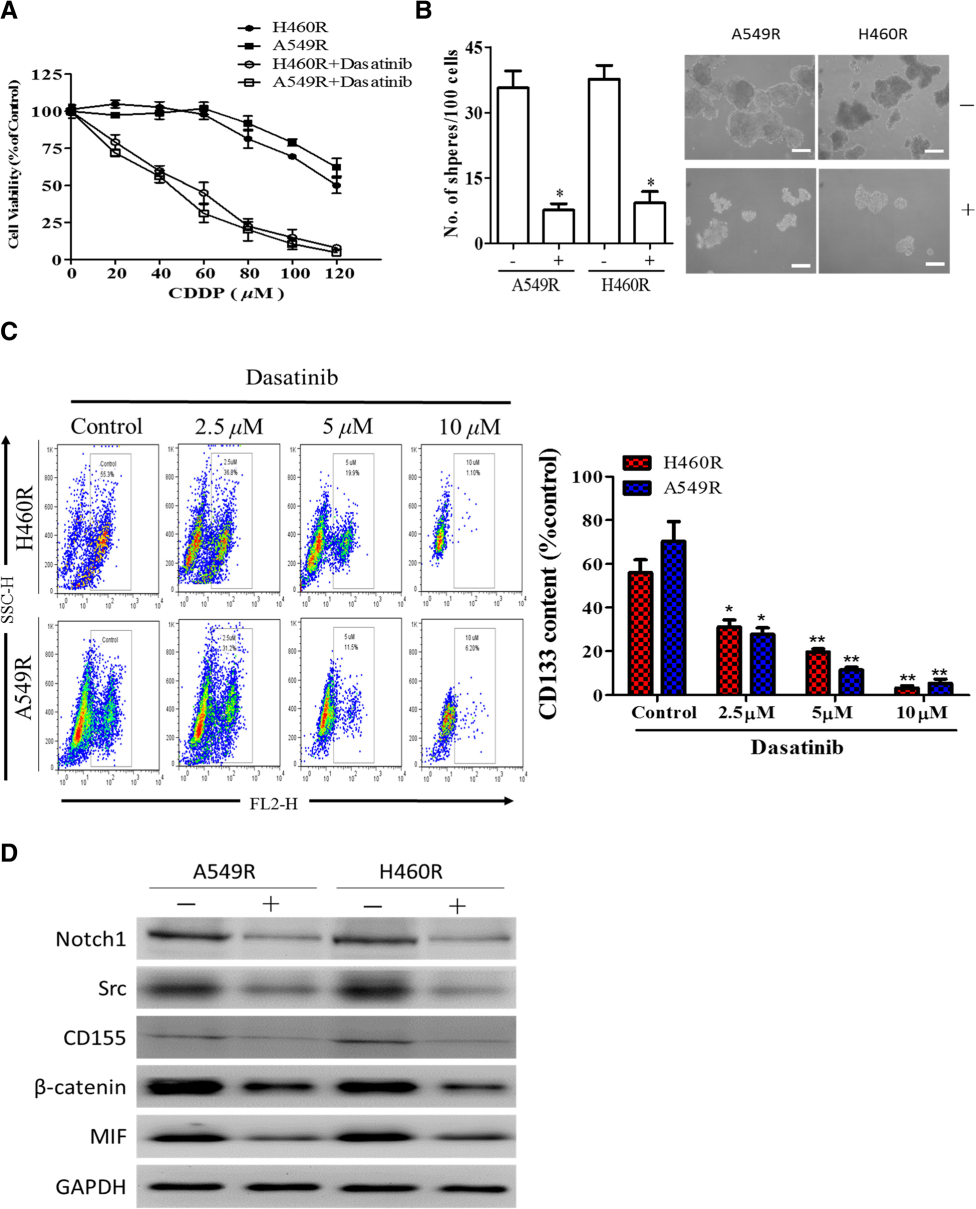
Dasatinib treatment decreased CDDP-resistance and stemness of NSCLC cells. **a** Comparative MTT assay showed that when A549R and H460R cells were first treated with dasatinib (1 μM, 24 h) followed by CDDP treatment demonstrated an increased sensitivity towards CDDP. **b** Tumor sphere formation assay showed that dasatinib treatment (10 μM) significantly inhibited the formation of tumor spheres in both A549R and H460R cells. **c** Flow cytometry analysis of CD133+ cells. Dasatinib treatment dose-dependently decreased the percentage of CD133+ cell population in both H460R and A549R cells. The bar graph on the right shows the comparative level of CD133+ cell population (from the flow cytometric analysis on the left) under different concentration of dasatinib treatment. *P < 0.05 as compared to the control. **d** Expression profiling of A549R and H460R post dasatinib treatment (5 μM, 24 h). The expression of pro-tumorigenesis molecules, Src, CD155 and MIF, cancer stemness molecules, Notch1 and β-catenin was all significantly suppressed under dasatinib treatment

Next, we examined the effect of dasatinib, a multiple kinase inhibitor, on both H460R and A549R cells. We pre-treated both H460R and A549R with dasatinib (1 μM, 24 h), followed by the treatment of cisplatin. We found that dasatinib treatment was able to significantly reduce the CDDP-resistance in both cell lines (Fig. 3a). For instance, the IC50 value of H460R against cisplatin decreased from > 120 to approximately 78 μM. In addition, dasatinib treatment significantly reduced the sphere forming ability in both H460R and A549R cells, approximately 4- and 3-fold respectively (Fig. 3b). Both the number and the size of the tumor spheres were significantly reduced under the treatment of dasatinib. We also examined the percentage of CD133+ cells (an established cancer stemness marker). In support, dasatinib treatment dose-dependently reduced CD133+ cell population in both CDDP-resistant NSCLC cell lines (Fig. 3c). As in the case of H460R cells, dasatinib treatment reduced the CD133+ cell population from 73.5 to 13.5% (approximately 5-fold decrease). The western blot analysis results corroborated that dasatinib treatment reduced the expression of stemness markers, Notch1, β-catenin, oncogenic markers, Src, MIF and CD155 (Fig. 3d). These observations suggest that dasatinib may be used for reducing tumorigenic and stemness properties of NSCLC cells.

### Dasatinib treatment reduced NSCLC induced M2 TAM polarization

**Fig. 4:**
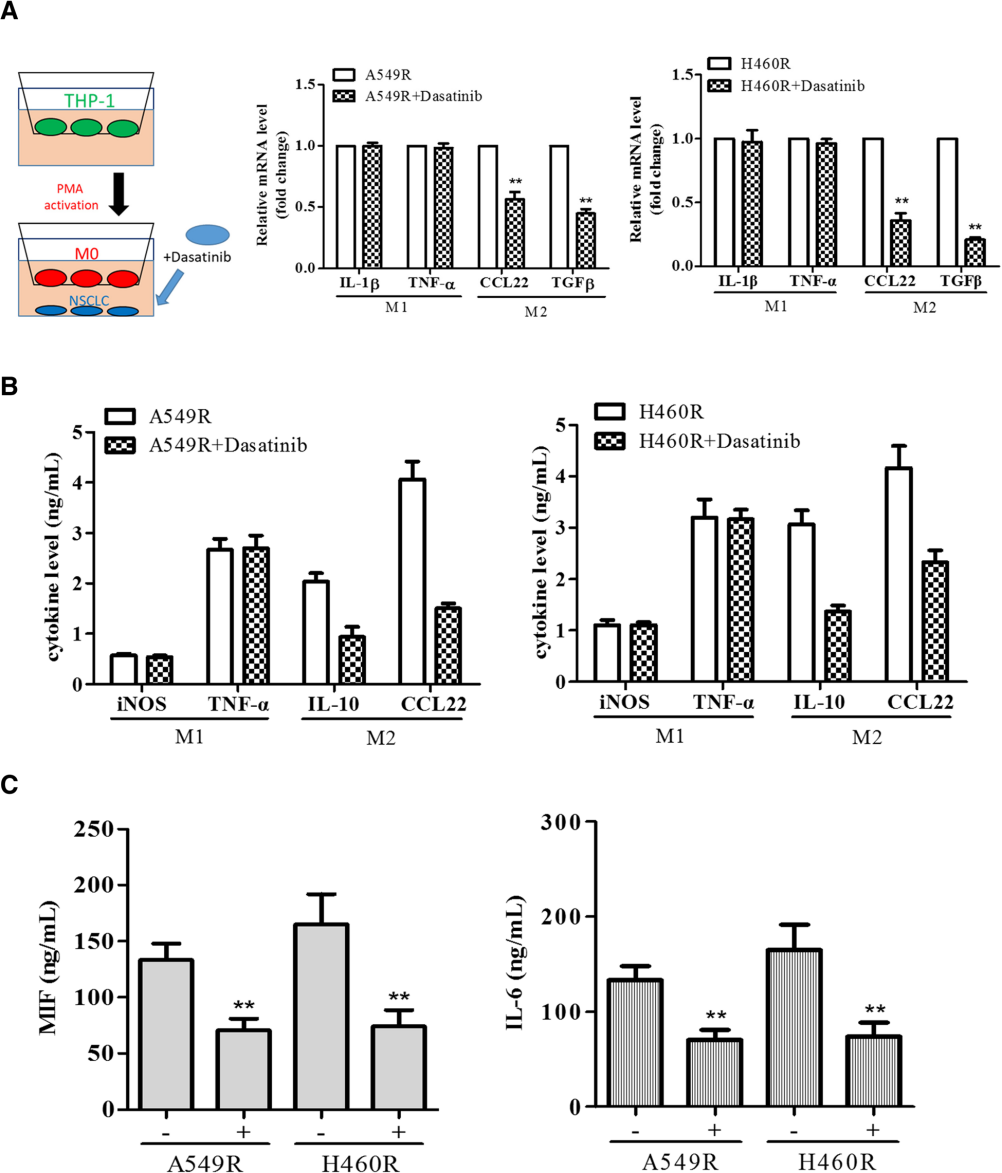
Dasatinib-treated NSCLC cells exhibited a lower ability to induce M2 polarization in macrophages. **a** Real-time PCR profiling of macrophages co-culture with NSCLC cells treated with dasatinib. qPCR results showed that the M2 markers, CCl13 and TGF-β were significantly reduced when co-cultured with H460R and A549R treated with dasatinib (2 μM, 24 h prior to co-culture), while M1 markers, IL-1β and TNF-α remained relatively unchanged. **b** Comparative ELISA assays of M1 M2 cytokines. Macrophages co-cultured with dasatinib-treated A549R and H460R cells showed a significantly lower secretion of M2 cytokines, IL-10 and CCL22 as compared to their counterparts which were co-cultured with A540R and H460R cells without prior dasatinib treatment. **c** ELISA assay showed that dasatinib treatment lowered the MIF and IL-6 secreted by both A549R and H460R cells

After establishing that dasatinib treatment led to reduced CDDP-resistance and stemness in H549R and H460R cells, we examined it effects on M2 polarization. Both H460R and A549R cells were treated with dasatinib (5 μM, 24 h) prior to co-culture with M0 macrophages. The resultant TAMs exhibited a significantly reduced M2 markers (CCL13 and TGF-β) while no significant changes in the markers of M1 TAMs (TNF-α and IL-1β) (Fig. 4a). In addition, the amount of secreted M2 cytokines, IL-10 and CCL2 was significantly reduced from the M2 TAMs (Fig. 4b) while M1 cytokines remained relatively unchanged. The ELISA assay showed that dasatinib treatment lowered the MIF and IL-6 secreted by both A549R and H460R cells (Fig. 4c).

### Silencing Src in CDDP-resistant NSCLC cells demonstrated similar cellular responses as in dasatinib treatment

**Fig. 5:**
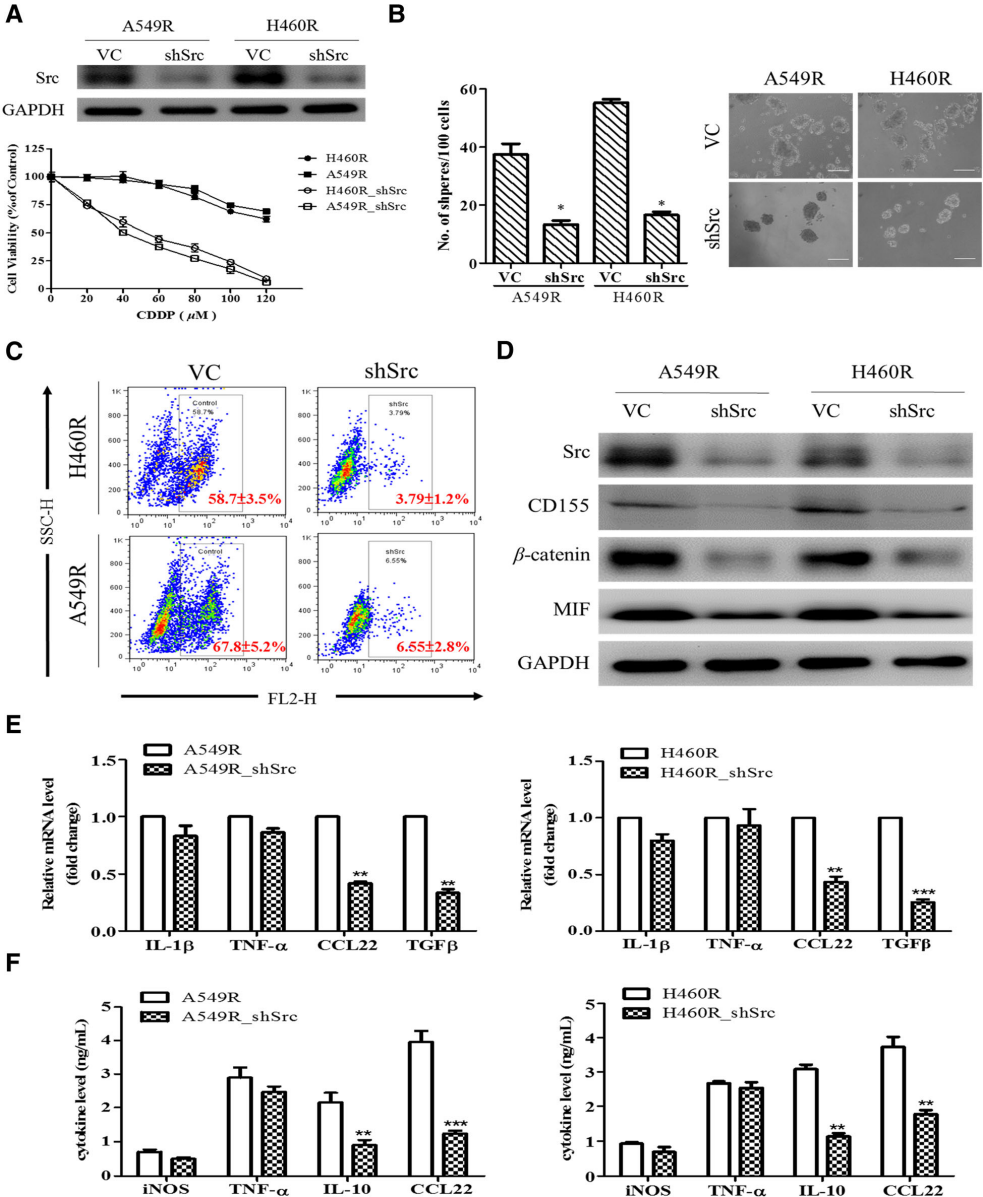
Silencing Src attenuated tumorigenic properties in H460R and A549R cells. **a** MTT assay showed that Src-silenced A549R and H460R cells became more sensitive towards CDDP treatment significantly as compared to their wildtype counterparts. **b** Src-silenced H460R and A549R cells showed a markedly reduced ability to form tumor spheres. **c** Aldefluor assay showed a significantly reduced ALDH1 activity in both A549R and H460R cells with Src-downregulated. The results are presented as mean ± SD. **d** Accompanied western blots showed that after Src was silenced in both cell lines, the expression of MIF, CD155 and β-catenin was clearly suppressed. VC, vector control; shSrch, Src-silencing by shRNA. **e** Real-time PCR results showed that mRNA level of M2 markers, CCL13, TGF-β was significantly down-regulated in the macrophage co-cultured with Src-silenced A549R and H460R cells. **f** ELISA assay showed that macrophages co-cultured with Src-silenced NSCLC cells secreted a significantly lower amount of M2 cytokines, IL-10 and CCL22, relative to their counterparts

Since Src tyrosine kinase is one of the main molecular targets of dasatinib, we examined the effects of silencing Src in both H460R and A549R cells. First, we found that Src-silenced H460R and A549R showed a significantly increased sensitivity towards cisplatin treatment. The IC50 values of H460R was decreased from > 120 to approximately 42 μM (Fig. 5a), which was almost back to its parental IC_50_ (approximately 40 μM, Fig. 1a). In addition, Src-silenced cells almost demonstrated a prominently reduced ability in forming tumor spheres (Fig. 5b). Silencing Src expression led to at least 2-fold reduction in tumor sphere generated in A549R cells (Fig. 5b). This observation was supported by the concomitant decreased percentage of ALDH1+ cells in both Src-silenced NSCLC cell lines (Fig. 5c). The protein expression of oncogenic markers, MIF, CD155 and β-catenin were all decreased as the result of Src-silencing (Fig. 5d). Furthermore, when co-cultured with macrophages, the resultant macrophages showed significantly decreased mRNA expression of M2 markers, CCL22 and TGF-β, in contrast to their wild-type counterparts (Fig. 5e); the amount of secreted M2 cytokines, IL-10 and CCL22 by the co-cultured macrophages was also marked decreased (Fig. 5f).

### Dasatinib treatment improved cisplatin sensitivity in A549R-bearing xenograft mice

**Fig. 6:**
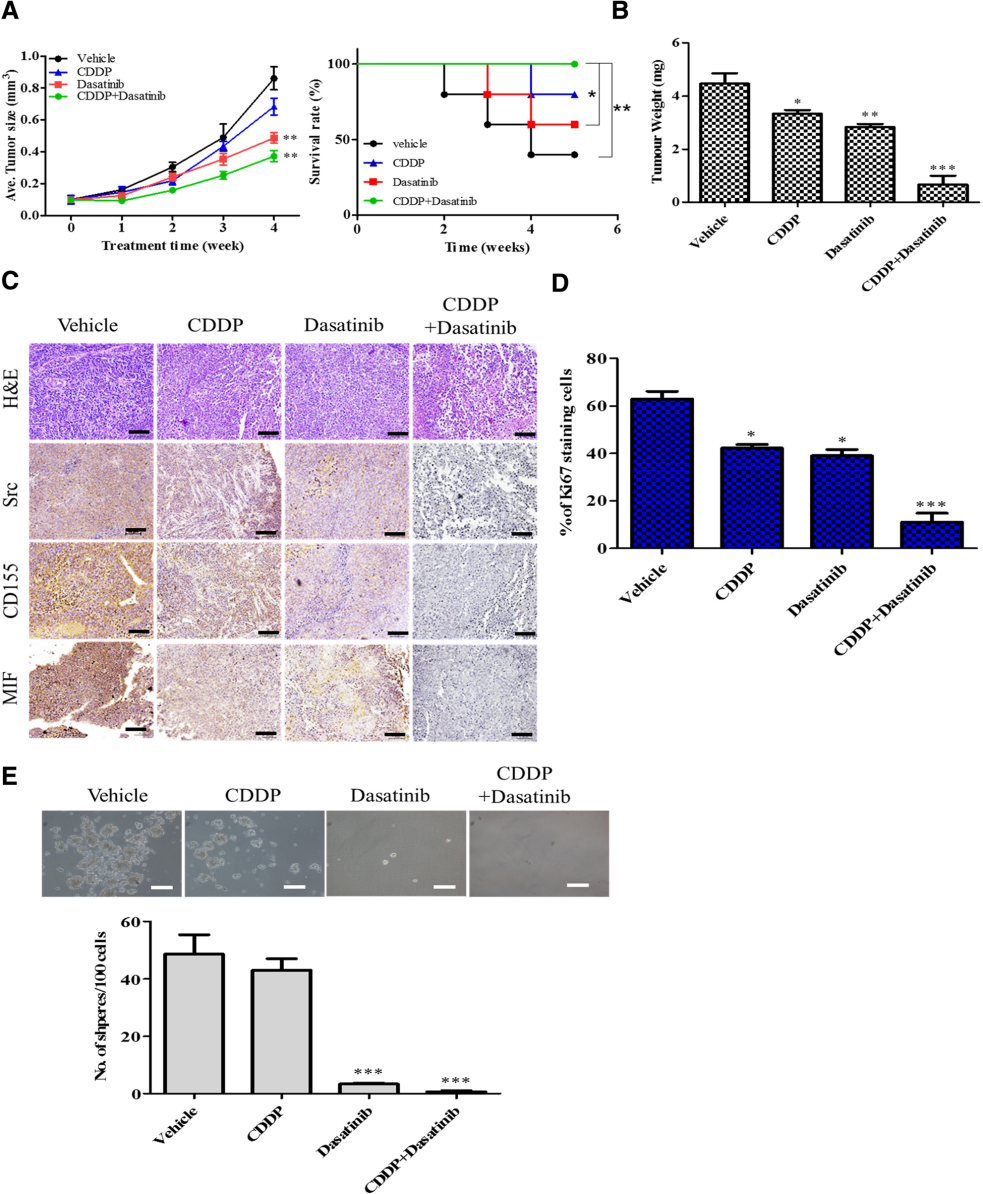
Dasatinib and Cisplatin combination suppressed drug-resistant lung cancer in vivo. **a** Tumor growth over time curve is depicted for A549R bearing mice with different treatment regimens, vehicle, cisplatin (CDDP), dasatinib and dasatinib+CDDP. The combination showed the lowest tumor burden followed by dasatinib. The tumor burden did not differ between the vehicle and CDDP groups. **p < 0.01 (as compared to the vehicle). **b** Survival curve and tumor weights are depicted. Significant differences between the treatment groups were made against the vehicle group. *p < 0.05; **p < 0.01 (as compared to the vehicle). **c** Immunohistochemical staining of sections showed that the combination treatment suppressed the expression of Src, MIF and CD133 to the most extent, followed by dasatinib and there was no significant difference between the vehicle control and CDDP alone groups. **d** Immunohistochemical staining of the nuclear proliferation marker Ki67. Percentage of Ki67-positive cells with significant differences compared to vehicle. **e** Tumor sphere formation comparative assay. The tumor cells from all groups were harvested and cultured under serum-free conditions. Tumor cells recovered from dasatinib+CDDP group showed the lowest ability of generating tumor spheres. **p < 0.01

Next, we transplanted A549R cells into NOD/SCID mice for validating the in vitro results. A549R-bearing mice were then subjected to different treatment regimens. We demonstrated that A549R cells in vivo also were resistant against cisplatin treatment, evident by the similar tumor growth curve as the vehicle control group (left panel, Fig. 6a). More importantly, the survival rate of the combination treatment group was significantly higher than the rest of the treatment groups (right panel, Fig. 6a). The tumor burden was the lowest in the cisplatin and dasatinib combined group, followed by the dasatinib alone group (Fig. 6a and b). Consistently, the immunohistochemical analysis of the tumor samples, the dasatinib and cisplatin combination group demonstrated the lowest staining intensity of Src, MIF, and CD155 (Fig. 6c). Consistently with the drop-in tumor volume and weight, co-treatment of mice resulted in a decrease in the number of Ki67+ cells (Fig. 6d). Notably, when the tumor cells were isolated and cultured under serum deprived conditions, the tumor cells from the combination group showed the lowest ability to generate tumor spheres as compared to all other groups (Fig. 6e).

### High expression of Src/CD155/MIF predicted poor survival in lung cancer patients

**Fig. 7:**
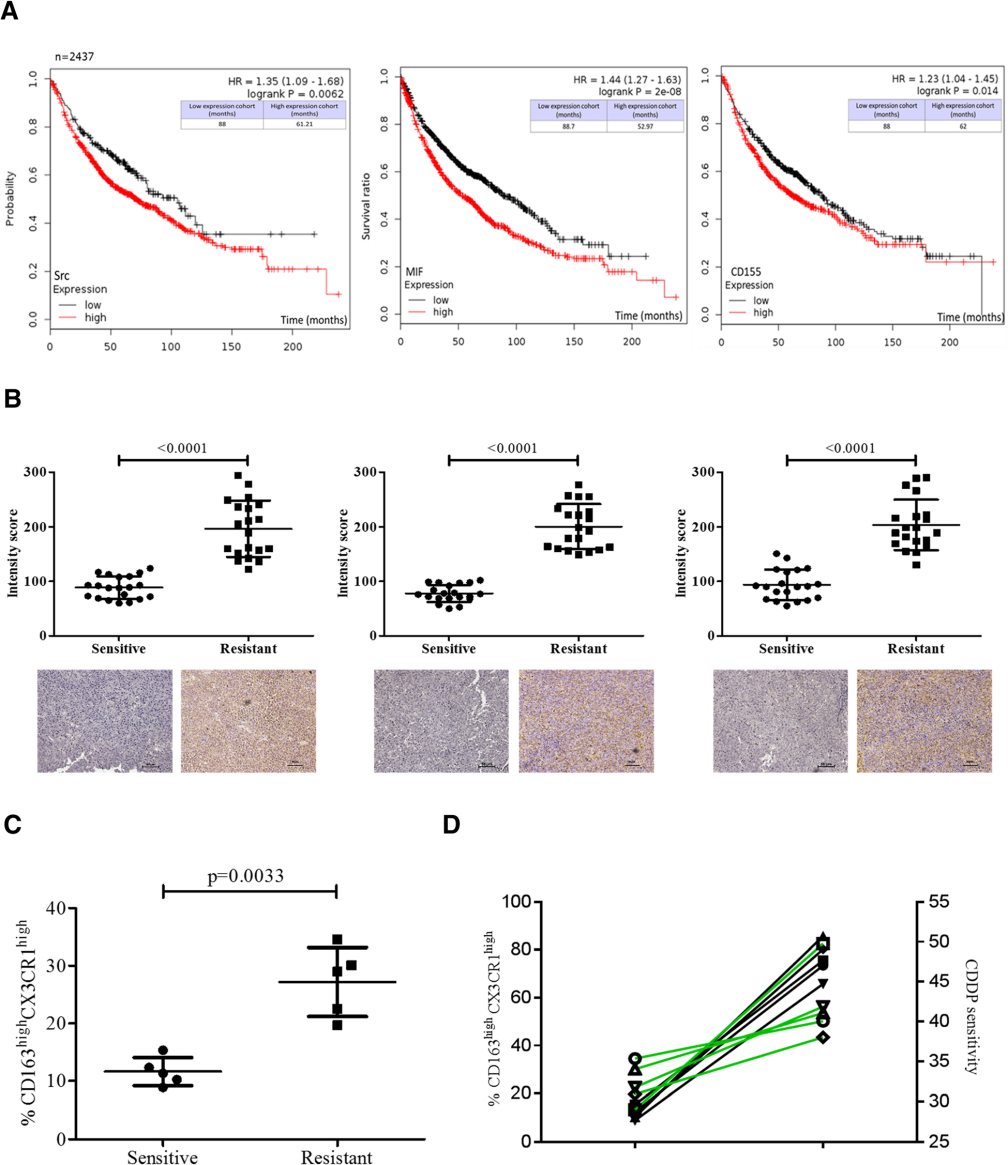
High expression of Src, MIF and CD155 is associated with poor survival in patients with NSCLC. **a** A public database of NSCLC patients was queried for Src, MIF and CD155 expression. Kaplan-Meier overall survival (KM) plots were drawn for each marker. The clinical samples were stratified into high and low expression of Src, MIF and CD155 respectively. In all three KM plots showed that the high expression of Src, MIF or CD155 predicts for a shorter overall survival rate in the patients. **b** Immunohistochemical analyses of 20 pairs clinical samples from our own hospital. The staining intensity score was compared between cisplatin sensitive and resistant samples. A higher staining score of Src, MIF and CD155 was observed in the resistant samples as compared to their cisplatin-sensitive counterparts. The lower panels depict the representative micrograph of samples stained with respective markers. *P*-value is indicated. **c** An increased CD163^high^ and CX3CR1^high^ M2 TAMs (by flow cytometry) were identified within the CDDP-resistant tumor samples as compared to CDDP-sensitive counterparts. **d** A negative correlation was found between the percentage of CD163^high^ and CX3CR1^high^ M2 TAMs and CDDP-sensitivity. The green lines delineate the connection between high percentage of CD163^high^ and CX3CR1^high^ M2 TAMs and their corresponding CDDP-sensitivity

In validation, we searched and analyzed public database (*n* = 2437) of lung cancer patients. The overall survival (OS) was calculated for Src, MIF and CD155 and these three markers all predicted a poor OS in patients with lung cancer (Upper panels, Fig. 7a). We validated with our own clinical samples using 20 pairs of tumor samples (sensitive versus resistant). The expression level of Src, MIF and CD155 (Fig. 7b) was significantly higher in the samples from patients non-responsive towards chemotherapeutics as compared to their counterparts. More importantly, we also demonstrated that the CDDP-resistant tumor samples contained a higher percentage of infiltrated M2 TAMs, indicated by a higher percentage of CD163^high^ and CX3CR1^high^ cells (Fig. 7c). More specifically, CDDP-resistant samples contained approximately 2.4-fold higher number of CD163^high^ and CX3CR1^high^ M2 TAMs as compared to CDDP-sensitive counterparts (Fig. 7c); equally important, a negative relationship was identified between the percentage of tumor infiltrated CD163^high^ and CX3CR1^high^ M2 TAMs and CDDP-sensitivity (Fig. 7d). The information on the determination of CDDP sensitivity and the characterization of clinical samples can be found in the Additional file 1: Table S1 and Additional file 2: Table S2.

## Discussion

Chemotherapy has been the standard treatment for all cancer types including the lung. However, accumulating evidence has indicated that chemotherapeutic agents may be one of the contributors for cancer cells to obtain resistance from selection pressure [12, 13]. More importantly, the tumor microenvironment also undergoes significant changes post chemotherapy. A previous study in cervical and ovarian cancer showed that cisplatin treatment was associated with an altered tumor microenvironment favoring the M2 polarization of macrophages. In this study, we first established cisplatin-resistant lung cancer cell lines, A549A and H460R, using escalating concentration of cisplatin over time. After CDDP-resistance was established, we found that these cells promoted M2 polarization in macrophages more prominently than their parental counterparts. Our observation added to the aforementioned study where chemotherapeutic agents such as CDDP, not only alters the microenvironment but also the tumor cells. Specifically, A549R and H460R cells exhibited stem cell-like properties, including increased CD133+ cell population, enhanced tumor sphere and colony forming abilities (Fig. 1). Thus, we have created a useful cellular platform for studying the underlying mechanisms by which lung cancer cells acquired CDDP resistance and their interactions with the TAMs.

Notably, the increased tumorigenic and stemness properties of A549A and H460R cells were accompanied by an increased in oncogenic markers such as Src tyrosine kinase, macrophage inhibitory factor (MIF), cancer stemness markers, Notch1 and β-catenin and multiple drug resistance genes, ABCG1 and ABCB1. Interestingly, MIF has recently been shown to be instrumental in promoting tumor growth and metastasis in breast cancer [14]. Another study demonstrated that breast cancer cells secreted MIF and led to the recruitment of S100A8+ myeloid cells, thereby promoted tumorigenesis [15]. Our data showed that there was a significantly increased level of MIF in both A549R and H460R cells when co-cultured with TAMs; as regards to macrophages, under the influence of A549R and H460R, TAMs exhibited a higher tendency towards M2 phenotype as evident by the increased M2 markers, CCL22 and IL-10 (Fig. 2). Equally important, oncogenic Src expression was significantly increased in the cisplatin-trained A549R and H460R cells, suggesting the elevated Src expression might be associated with the survival of the NSCLC cells in response to cisplatin exposure. Src tyrosine kinase has been recognized as one of the key drivers for many cancer types including lung cancer [16]. Recent evidence has shown that an increased Src tyrosine kinase signaling is associated to increased metastatic potential and cancer stemness [17, 18]. These reports support our observation that cisplatin-resistant A549R and H460R exhibited an increased Src expression as compared to their parental counterparts (Fig. 3). An elevated Src/β-catenin signaling was associated with increased metastatic potential in both A549R and H460R cells was supported by a previous study [19]. Collectively, cisplatin resistant A549R and H460R cells provided some insights into cisplatin-induced drug resistance and stemness in lung cancer. Our data strongly suggests that mono-therapy with CDDP may not be beneficial to patients with lung cancer but rather disadvantageous for survival due to its potential risk of enriching cancer stemness.

With the heterogenous tumor microenvironment, tumor associated macrophages (TAMs) have been shown to infiltrate into the tumor mass and promote tumorigenesis by interacting intimately with tumor cells. M2 polarized TAMs or M2 TAMs especially received the most attention since they are one of the key contributors to angiogenesis, epithelial-mesenchymal-transition (EMT), and distant metastasis [20, 21]. Our co-culture experiments provided interesting observations where A549R and H460R NSCLC cells were more capable of promoting M2 polarization of TAMs than their parental counterparts. More specifically, these A549R and H460R cells were generated in simulation of clinical settings where continuous chemotherapeutic agents were administered. The significantly increased Src expression in both A549R and H460R cells were supported by previous study where Src was activated to support the survival and migration of afatinib-resistant lung cancer cells [22]. Our study adds new insights that cisplatin-resistant A540R and H460R cells promoted the generation of M2 TAMs via the increased expression of pro-M2 cytokines, IL-4, IL-10 and MIF. The generation of M2 TAMs may subsequently facilitate the initiation and development of distant metastasis (Fig. 4). Our findings offer additional dimension for therapeutic development since we focus on both tumor cells and their microenvironment.

Of interest, recently CD155 was reported to be elevated in the sera of cancer patients as compared to normal sera; the elevated serum CD155 level was linked to the progression of lung cancer [4]. Further support indicated that an elevated CD155 expression not only was identified on the tumor cells but also on the tumor infiltrating myeloid cells [23]. These observations provide strong support to our observations where an increased CD155 expression was detected on both H460R and A549R cells; more importantly, the elevation of CD155 was in association with increased level of Src and stemness markers. In turn, M2 polarization of TAMs was significantly enhanced. Uniquely, our observations shed some light on the complex signaling networks which were activated for the survival of lung cancer cells under CDDP assault. We are currently investigating the regulatory mechanisms of CD155 in lung cancer cells as well as in other immune cells (Fig. 5).

To translate our finding towards future clinical settings, we showed that the suppression of Src either by dasatinib treatment or gene silencing, the aforementioned tumorigenic effects were significantly inhibited. We showed that the suppression of Src in both A549R and H460R cells was associated with a significantly reduced oncogenic and stemness properties. This is in tune with a previous study where the usage of Src inhibitor led to the suppression of cell growth and apoptosis induction in gefitinib-resistant NSCLC cells [24]. Our experimental data provided further evidence to support the usage of Src inhibitor, dasatinib, or silencing of Src led to the decreased ability of A549R and H460R cells to promote M2 polarization of TAMs. Finally, our in vivo results showed that the combination of cisplatin and dasatinib significantly suppressed the tumor growth in A549R xenograft mouse model and the combination treatment led to the best survival rate among different groups (Fig. 6). This finding provided a strong support to our hypothesis that Src suppression could re-sensitize cisplatin-resistant lung cancer cells. We also found that tumor cells harvested from the combination group, showed a lowest ability for generating tumor spheres. This observation suggests that the combination of dasatinib and cisplatin could prevent the generation of cancer stem cells and perhaps prevent tumor recurrence. We then established that the high expression level of Src, MIF, or CD155 predicted a poor overall survival in the patients with NSCLC using a public database consisting of 2437 patients [9]. In support, our own clinical samples which were tested for their cisplatin sensitivity (sensitive versus resistant), corroborated with the database prediction (Fig. 7). Moreover, a higher percentage of M2 TAMs (CD163^high^CX3CR1^high^) cells were found infiltrated within the CDDP-resistant lung cancer samples as compared with their CDDP-sensitive counterparts (Fig, 7C and D). Collectively, our findings along with others strongly suggest the combination of Src inhibitor with current chemo- and/or targeted therapeutic agents may reduce the possibility of drug resistance and disease progression. It is worth noting that there are ongoing clinical trials using dasatinib alone or in combination with other targeted agents. The results of our study may provide additional information for designing future clinical trials which target Src signaling pathways.

## Conclusions

**Fig. 8:**
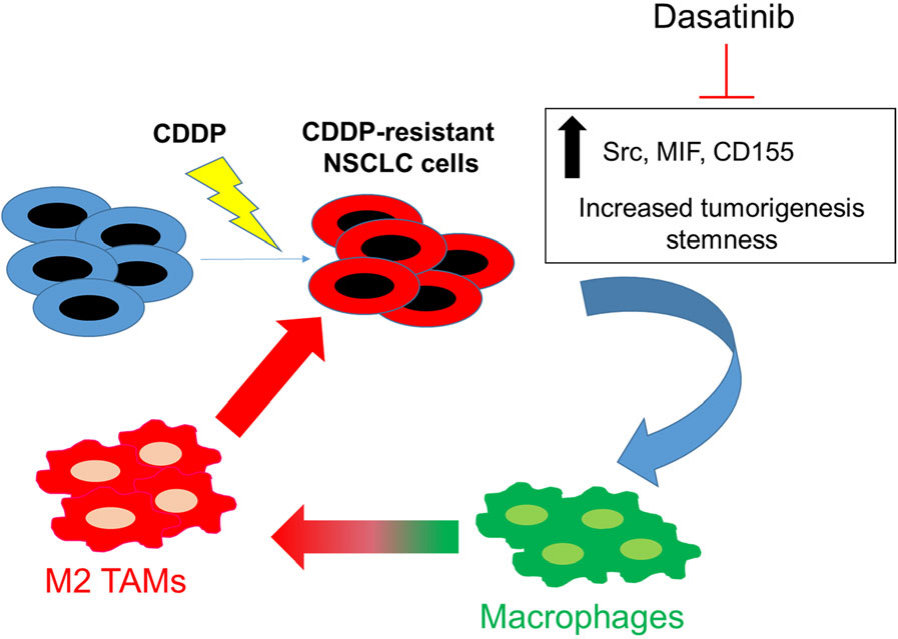
Schematic diagram of cisplatin resistant lung cancer cells promoted M2 polarization of tumor-associated macrophages via the Src/CD155/MIF functional pathway. Thus, kinase inhibitors such as dasatinib may be of potential for treating cisplatin-resistant lung cancer by targeting both tumor and the tumor microenvironment

In summary, as depicted in the schematic abstract of Fig. 8 provided important insights into the development of cisplatin resistance in lung cancer cells. Cisplatin-resistant A549R and H460R cells showed increased stemness properties and ability to modulate the tumor microenvironment, specifically in the generation of M2 TAMs. These increased pro-tumor properties were associated with a substantially increased expression in Src/CD155/MIF expression and stemness markers such as Notch1 and β-catenin. The suppression of Src signaling led to the dampening signals in CD155, MIF, Notch1 and β-catenin. Thus, it may be worthy of pursuing the combination of Src inhibitor with current chemotherapeutic agents for treating NSCLC patients with failed prior treatments.

## Additional files

Additional file 1: **Table S1.** CDDP sensitivity assay of tissue samples from lung cancer patients (please refer to Additional file 2: Table S2 for patients’ characteristics). (DOCX 17 kb)

Additional file 2: **Table S2.** Pathological characteristics of patients. (DOCX 14 kb)

Additional file 3: **Figure S1.** Prolonged, escalating CDDP treatment enriched CDDP-resistant H460R and A549R NSCLC cells. MTT assay demonstrated that H460R and A549R cells with a significantly higher IC50 values against CDDP treatment. (DOCX 19 kb)

## Abbreviations

5-FU: 5-flurouracil
CDDP: Cisplatin
CSCs: Cancer stem cells
DTX: Docetaxel
ECM: Extracellular matrix
EMT: Epithelial-to-mesenchymal transition
FBS: Fetal bovine serum
IFC: Immunofluorescence
SRB: Sulforhodamine B
TAMs: Tumor associated macrophages
TCA: Trichloroacetic acid
TICs: Tumor-initiating cells
TME: Tumor microenvironment
