# Supplementary material for: Cisplatin resistant lung cancer cells promoted M2 polarization of tumor-associated macrophages via the Src/CD155/MIF functional pathway
Source: J Exp Clin Cancer Res. 2019 Apr 29;38:180. doi: 10.1186/s13046-019-1166-3 (PMC6489343; doi:10.1186/s13046-019-1166-3)
Supplement: Supplementary file 1 — Table S1. CDDP sensitivity assay of tissue samples from lung cancer patients (please refer to Additional file 2: Table S2 for patients’ characteristics). (DOCX 17 kb) [file 13046_2019_1166_MOESM1_ESM.docx]

**Supplementary Materials and Methods**

**Chemo-sensitivity test**

We defined CDDP-sensitivity using the succinate dehydrogenase inhibition (SDI) assay as developed and described previously [1, 2]. Briefly, after removing necrotic tissues, the tumor tissue was processed in McCoy's 5A solution supplemented with antibiotics, pronase (Sigma Chemical, USA), 0.1% collagenase (type I, Sigma) and DNase I (type I, Sigma). The enzymatic dissociation process was carried out for 20 min at 37°C, and was stopped by adding minimal essential medium (MEM). Cells were then passed through a nylon mesh, rinsed with MEM, pelleted, and resuspended. Single cell suspension was subsequently dispensed into 96‐well microtiter plates, and incubated at 37°C for 3 days in the presence of cisplatin (CDDP, 20μg/ml). Under this CDDP concentration, the SD activity was reported to reflect the cell viability optimally. Post CDDP treatment, MTT assay was carried out and the absorbance at 540 nm was quantitated using a microtiter plate reader. The SD activity was presented as the optical density per milligram of protein. The chemo-sensitivity to CDDP was indicated as a percentage of the SD activity in CDDP- treated cells over the control cells. CDDP sensitivity rate (%) = 100‐(Treatment ‐blank)/(Control‐blank) ×100% (Treatment: absorbance of tumor cells exposed to CDDP; control, untreated cells: absorbance of untreated cells; blank: baseline absorbance).

**Supplementary Table 1.** CDDP sensitivity assay of tissue samples from lung cancer patients (please refer to Supplementary Table 2 for patients’ characteristics)

|  | CDDP sensitivity rate | | P value |
| --- | --- | --- | --- |
| CDDP | 52.3 ± 10.4 | 73.4 ± 14.0 | 0.02 |

A higher number of CDDP sensitivity rate indicates the cells are more sensitive towards CDDP treatment. In this case, the group 73.4 ± 14.0 is more sensitive towards CDDP treatment than the group 52.3 ± 10.4.

**References:**

1. Oki E, Baba H, Tokunaga E, Nakamura T, Ueda N, Futatsugi M, Mashino K, Yamamoto M, Ikebe M, Kakeji Y, Maehara Y. Akt phosphorylation associates with LOH of PTEN and leads to chemoresistance for gastric cancer. Int J Cancer. 2005; 117: 376-80. doi: 10.1002/ijc.21170.
2. Shibuya K, Mathers CD, Boschi-Pinto C, Lopez AD, Murray CJ. Global and regional estimates of cancer mortality and incidence by site: II. Results for the global burden of disease 2000. BMC Cancer. 2002; 2: 37. doi:
