# Supplementary material for: Cisplatin resistant lung cancer cells promoted M2 polarization of tumor-associated macrophages via the Src/CD155/MIF functional pathway
Source: J Exp Clin Cancer Res. 2019 Apr 29;38:180. doi: 10.1186/s13046-019-1166-3 (PMC6489343; doi:10.1186/s13046-019-1166-3)
Supplement: Supplementary file 2 — Table S2. Pathological characteristics of patients. (DOCX 14 kb) [file 13046_2019_1166_MOESM2_ESM.docx]

**Supplementary Table 2.** Pathological characteristics of patients

| Categories | All patients (n = 40) |
| --- | --- |
| Age |  |
| <65yr | 12 (30%) |
| ≥65yr | 28 (70%) |
| Sex |  |
| Male | 30 (75%) |
| Female | 10(25%) |
| Smoking status |  |
| PYI<20 | 10 (25%) |
| PYI ≥ 20 | 30 75%) |
| Histological type |  |
| Squamous | 12 (30%) |
| Adeno | 28 (70%) |
| Differentiation |  |
| Well | 9 (23.5%) |
| Moderate | 14 (35%) |
| Poorly | 17 (42.5%) |
| p‐TNM stage |  |
| StageI | 15(37.5%) |
| StageII | 11 (27.5%) |
| StageIII | 14 (35%) |
| Pleural invasion |  |
| Yes | 28 (70%) |
| No | 12(30%) |
| Vascular invasion |  |
| Yes | 24 (60%) |
| No | 16 (40%) |
| Lymphatic invasion |  |
| Yes | 35(87.5%) |
| No | 5 (12.5%) |
